# A [3]Rotaxane Containing {Ti_7_Ga} Rings Linking Cu^II^: Synthesis, Structure, and Spectroscopic Studies

**DOI:** 10.1002/chem.202502530

**Published:** 2025-09-30

**Authors:** Selena J. Lockyer, Lubomir Loci, George F. S. Whitehead, Inigo J. Vitorica‐Yrezabal, Grigore A. Timco, Alice M. Bowen, Eric J. L. McInnes, Richard E. P. Winpenny

**Affiliations:** ^1^ Department of Chemistry and Photon Science Institute The University of Manchester Oxford Road Manchester M13 9PL UK

**Keywords:** DEER/PELDOR, EPR, heterometallic rings, rotaxanes, supramolecular

## Abstract

We report the synthesis of extended hybrid organic‐inorganic rotaxanes based on (pyCH_2_NH_2_CH_2_CH_2_py)[Ti^IV^_7_Ga^III^O_8_(O_2_C^t^Bu)_16_] (**1**) and (pyCH_2_NH_2_CH_2_py)[Ti^IV^_7_Ga^III^O_8_(O_2_C^t^Bu)_16_] (**3**) building blocks, containing an anionic macrocycle and doubly pyridyl‐terminated secondary ammonium threads. Reaction of **1** and **3** with [Cu(hfac)_2_] (hfac = 1,1,1,5,5,5‐hexafluoroacetylacetonate) gives {[Cu(hfac)_2_]_3_[**1**]_2_} (**2**) and {[Cu(hfac)_2_]_2_[**3**]} (**4**), respectively, which are characterized by single‐crystal X‐ray crystallography. The products are controlled by the differing steric demands imposed by the two arms of the thread. The structure of **2** is an extended [3]rotaxane, with two molecules of **1** binding to a central [Cu(hfac)_2_] molecule via the longer arm of the thread, and two [Cu(hfac)_2_] units terminating the structure at the shorter arm. The structure of **4**, where both arms of the thread are short, is an extended [2]rotaxane terminated by two molecules of [Cu(hfac)_2_]. The Cu…Cu…Cu fragment in **2** is linear, with the central Cu lying on an inversion center and with a separation between the terminal copper ions of 31.3 Å. Double Electron‐Electron Resonance (DEER; also known as PELDOR) spectroscopy proves that the [3]rotaxane structure remains intact in solution, detecting both terminal…center and terminal…terminal Cu…Cu interactions, with orientation‐selective measurements demonstrating that there is a rearrangement of the structure at the terminal positions on dissolution.

## Graphical abstract

Extended hybrid inorganic‐organic [2]‐ and [3]‐rotaxanes are reported based on heterometallic rings with threads that link Cu^II^ complexes; the crystal structures are reported, and the solution behavior is investigated by double electron electron resonance spectroscopy methods.

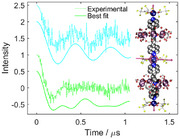

## 1 Introduction

A number of groups are studying electron spins within molecules as potential quantum bits (qubits) for use in quantum information processing.^[^ ^1^, ^2^ ^]^ Most groups study organic radicals^[^ ^3^ ^]^ or monometallic coordination compounds.^[^ ^4^, ^5^, ^6^, ^7^, ^8^, ^9^ ^]^ A minority study polymetallic compounds, for example, the Aromí group, which has worked on heterometallic lanthanide complexes^[^ ^10^, ^11^ ^]^ and more recently on Cu^II^‐Ni^II^ complexes.^[^ ^12^ ^]^ Increasingly, groups are attempting to link together qubits^[^ ^3^, ^12^, ^13^, ^14^, ^15^ ^]^ as no useful algorithm can be performed with a single qubit.

Our work has focused on the heterometallic rings {Cr^III^_7_Ni^II^},^[^ ^2^, ^13^, ^16^, ^17^ ^]^ which have an *S* = 1/2 ground state and are therefore a two‐level system. These rings have a disadvantage in that their coherence times^[^ ^18^ ^]^ tend to be shorter than those in simpler molecules, but they gain advantages from the complexity of the structures, which allows them to be linked into multiple qubit arrays.^[^ ^13^, ^19^, ^20^, ^21^, ^22^, ^23^ ^]^ A particularly attractive example is a five‐qubit supramolecule involving two {Cr^III^_7_Ni^II^} and three Cu^II^ complexes, with two distinct interaction energies between the qubits.^[^ ^24^ ^]^ This led us to propose these could be used to study coherence in strongly entangled states.

The synthetic chemistry intrigues us, particularly as the presence of sixteen carboxylate ligands tunes the solubility of complexes across a wide range of solvents. We have made many further examples of general formula [Cation][M_7_M'E_8_(O_2_CR)_16_], where the cation is typically a secondary ammonium cation and R can be almost any side‐chain, for example, ‐^t^Bu, ‐CH_2_^t^Bu, ‐C_6_H_5_, ‐C_4_H_8_S. This has included rings where E = F, which gives rings with M = Fe^III^, V^III^, Ga^III^, In^III^, Al^III^, and M’ = Ni^II^, Co^II^, Zn^II^, Cd^II^, Mn^II^, Cu^II^, Mg^II^. We have also reported cases where E = O and M = Ti^IV^, with M’ = Fe^III^, V^III^, Ga^III^, In^III^, Al^III^, Ti^III^.^[^ ^25^ ^]^ Here we report a new molecule where we used the diamagnetic {Ti^IV^_7_Ga^III^} ring to bridge between individual Cu^II^ sites. By controlling the distance between the Cu^II^ sites, we can predict the outcome of the supramolecular adduct. Hence, by design we find a discrete molecule containing three *S* = 1/2 Cu^II^ complexes linked by two rings.

## 2 Results and Discussion

### 2.1 Synthesis and Structures

**Figure 1:**
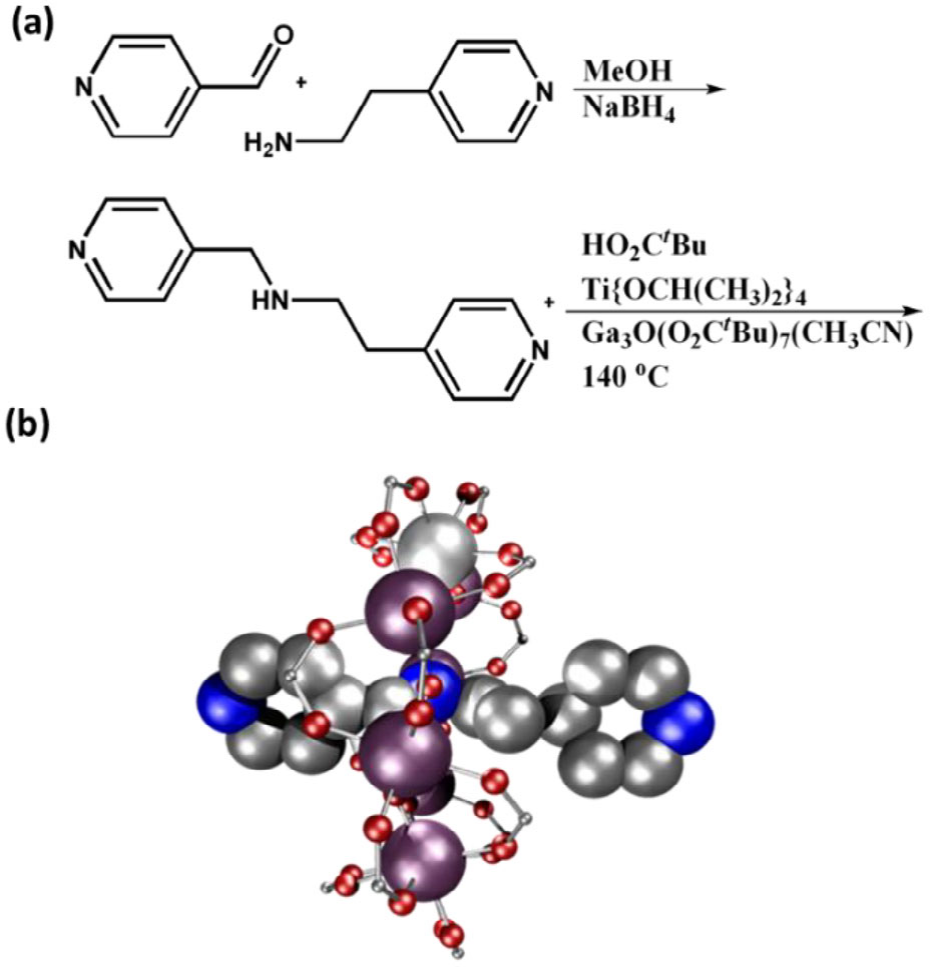
a) The synthesis and b) the structure of **1**. Atoms within the thread **A**H^+^ are shown with Van der Waals radii. All other atoms are shown as ball‐and‐stick representations. Atom colors: blue (N), red (O), grey (C), mauve (Ti), silver (Ga). *^t^* Bu groups and hydrogens omitted for clarity.

We first synthesized an amine that contains two pyridyl groups: pyCH_2_NHCH_2_CH_2_py, **A**. **A** can be straightforwardly made from a Schiff‐base condensation followed by a reduction (Figure 1a; see Supporting Information for experimental details).^[^ ^26^ ^]^ This can then be reacted with [Ga_3_O(O_2_C*^t^* Bu)_7_(MeCN)] and [Ti(O*^i^* Pr)_4_] to give (**A**H)[Ti_7_GaO_8_(O_2_C*^t^* Bu)_16_] **1** (Figure 1b). The threads are held in place by coulombic forces between the secondary ammonium cation **A**H^+^ and the anionic heterometallic ring [Ti_7_GaO_8_(O_2_C*^t^* Bu)_16_]^−^, and also by hydrogen bonding between the ammonium protons and the oxides on the interior of the ring.^[^ ^16^, ^17^ ^]^ Although threading/de‐threading reactions are possible with pre‐made rings,^[^ ^27^ ^]^ the direct syntheses are believed to proceed via templating. We have been unable to synthesize a {Cr_7_Ni} ring templated about **A**H^+^. It is possible to make [PhCH_2_CH_2_NH_2_CH_2_py][Cr_7_NiF_8_(O_2_C^t^Bu)_16_];^[^ ^28^ ^]^ there is no obvious explanation for this difference between the {Ti_7_Ga} and the {Cr_7_Ni} rings.

**Figure 2:**
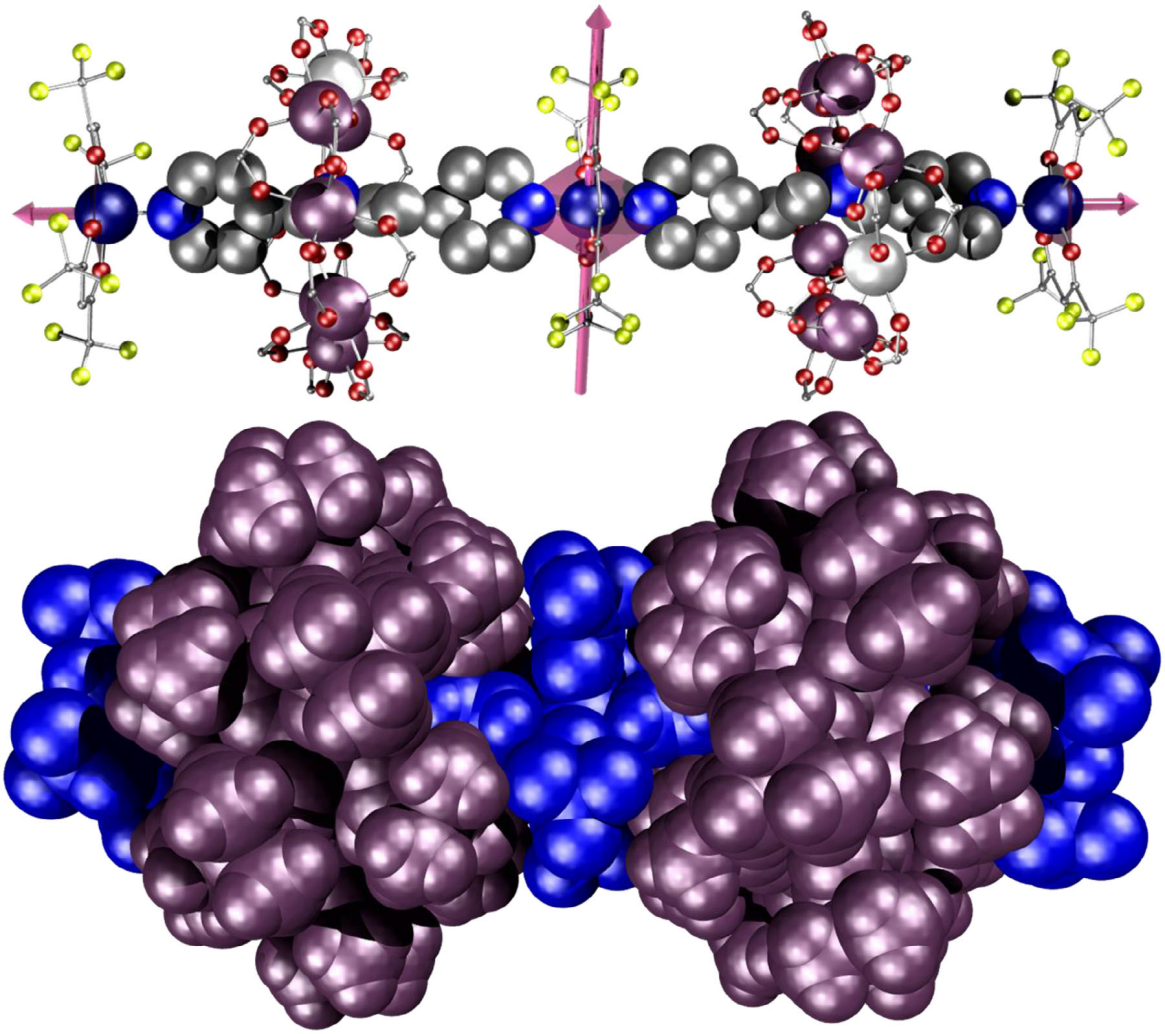
Top: The structure of **2** in the crystal. Atom colors as per Figure 1 with yellow (F) and navy blue (Cu) atoms. The purple vectors show the direction of the local magnetic *z*‐axes, and the purple planes show the local magnetic *xy*‐planes at each Cu^II^ site in the crystal structure. Bottom: space‐filling representation with threads and [Cu(hfac)_2_] units shown in blue, and {Ti_7_Ga} rings in mauve.

Reaction of **1** with 1.5 equivalents of [Cu(hfac)_2_] (where hfac = 1,1,1,5,5,5‐hexafluoroacetylacetonate), under an inert atmosphere and in a solution of dry Et_2_O and toluene, produces {[Cu(hfac)_2_]_3_[**1**]_2_} **2** (Figures 2, top, and S2). Compound **2** crystallizes with the central Cu^II^ ion on an inversion center and contains two molecules of **1** and three of [Cu(hfac)_2_].^[^ ^29^ ^]^ The asymmetric thread allows two molecules of **1** to bind to the central [Cu(hfac)_2_] through the pyridyl attached to the ethylene group of **A**H^+^. The pyridyl bound to the methylene group of **A**H^+^ makes this end more sterically demanding, and only one can be attached to a [Cu(hfac)_2_] unit because it is pulled into the region of the bulky ^t^Bu groups of the pivalate ligands on the exterior of the ring (Figure 2, bottom). (This is confirmed by the structure of compound **4**; see below.)

Compound **2** is a [3]rotaxane, with the thread made up of two **A**H^+^ cations and the three [Cu(hfac)_2_] units. The distance between the two terminal Cu^II^ positions (Cu1) is 31.3(1) Å. The center of each {Ti_7_Ga} ring is 7.32(1) Å from its respective Cu1 and 8.40(1) Å from the central Cu site (Cu2), and the linear distance between metal cores of the two {Ti_7_Ga} rings is 16.8(1) Å. The two terminal [Cu(hfac)_2_] groups are five‐coordinate with a square pyramidal geometry (τ_5_ = 0.00145). The pyridyl nitrogen is at the apex, with a Cu‐N bond length = 2.19(1) Å. The four Cu…O bond lengths are 1.94(1) and 1.99(1) Å; symmetry means there are only two unique distances. This structure would place the local *z*‐axis for the Cu1 electronic ***g***‐matrix parallel to the Cu1…Cu2…Cu1 axis, where the latter is linear as enforced by the inversion center at Cu2.

The central [Cu(hfac)_2_] group is six‐coordinate with an octahedral geometry. Two molecules of **1** bind *trans‐* to one another about the Cu^II^ ion with N^py^…Cu bonds of 2.05(1) Å. The Cu‐O bonds are distinct, with two long O…Cu bonds of 2.24(2) Å, and two short O…Cu bonds of 1.96(1) Å. This indicates that the Jahn‐Teller axis (hence the local *g*_z_ axis) at the Cu2 lies along one of the O‐Cu‐O directions. It is therefore perpendicular to the unique axes at the terminal Cu^II^ sites, which lie along the N‐Cu direction (Figure 2, top).

The Ga^III^ ion is delocalized across all eight metal ion positions of each {Ti_7_Ga} ring. The metal sites within the ring are all six‐coordinate, with distorted octahedral geometries. The M─O(oxide) bonds fall in the range 1.80–1.85(1) Å, while the M‐O(carboxylate) bonds are in the range 2.00–2.07(1) Å. The protonated secondary ammonium of **A**H^+^ forms H‐bonds to the bridging oxides of the {Ti_7_Ga} ring with the shortest N‐H…O distance being 2.86(1) Å.

To complement the structural results of **2**, a symmetrical equivalent of the diamine thread **A** was prepared; pyCH_2_NHCH_2_py (**B**), where both links to the pyridyls are the shorter methylene link. **B** can be reacted with [Ga_3_O(O_2_C*^t^* Bu)_7_(MeCN)] and [Ti(O*^i^* Pr)_4_] to give the symmetrical [2]rotaxane (**B**H)[Ti_7_GaO_8_(O_2_C*^t^* Bu)_16_] **3** (Figure S1). Reaction of **3** with two equivalents of [Cu(hfac)_2_], under an inert atmosphere and in a solution of dry Et_2_O and toluene, produces {[Cu(hfac)_2_]_2_^[^ **^3^** ^]^} **4**. The symmetric thread is sterically demanding on both ends, such that compound **4** crystallizes with only two Cu^II^ ions, one terminating at each of the pyridyls of **B**; hence, **4** contains only one molecule of **3** and two [Cu(hfac)_2_] (Figure S3).^[^ ^28^ ^]^

Compound **4** is a [2]rotaxane, containing two terminal Cu^II^ ions with a separation of 14.3(1) Å between them. The distance from the center of the {Ti_7_Ga} ring to each Cu^II^ ion is 7.19(1) Å. The [Cu(hfac)_2_] groups show different geometries to that of **2** and are disordered within the crystal structure. The Cu^II^ ions are five‐coordinate and square pyramidal (τ_5_ ranges from 0.0213 to 0.1033 due to disorder in the crystal structure). In contrast to **2**, the pyridyls bind in an equatorial site of the square pyramid, with one of the Cu…O bonds defining the apical site. We have seen such a geometry before in [Cu(hfac)_2_]{(pyCH_2_NH_2_CH_2_CH_3_)[Cr_7_NiF_8_(O_2_C^t^Bu)_16_]}.^[^ ^22^ ^]^ Hence, in this conformation, the Cu coordination geometry is rotated by 90 degrees with respect to that of the terminal Cu1 ions in **2**.

### 2.2 CW EPR Spectroscopy

**Figure 3:**
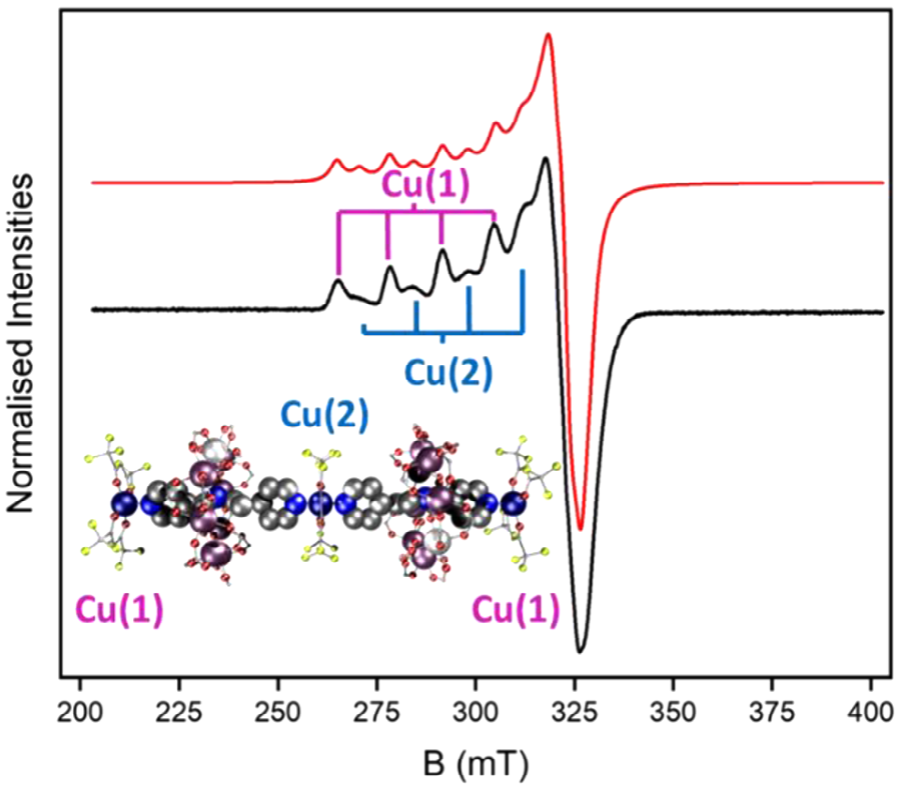
X‐Band (ca. 9.5 GHz) cw EPR spectra of **2**, as a 1 mM solution in Et_2_O. Measured at 5 K (black line) and simulated with parameters in the text (red line).

Continuous‐wave (cw) X‐band (ca. 9.5 GHz) EPR spectroscopy measurements were performed on **3**, as a powder and a 1 mM solution in dry Et_2_O (Figures 3 and S4). Because the {Ti^IV^_7_Ga^III^} rings are diamagnetic, the EPR response is only due to the Cu^II^ ions. At 5 K, spectra in both powder and frozen solution measurements show well‐resolved EPR signals typical of Cu^II^, which are consistent with near‐axial *g*‐matrices and ${\left(d_{x^{2}-y^{2}}\right)}^{1}$ ground state configurations (*xyz* refer to the local axes at each Cu). The linewidth of the frozen solution spectrum is sharp enough that two sets of quartet peaks are seen in the *g*_z_ part of the spectrum, with a relative intensity of 2:1. Hence, the hyperfine interactions with the ^63,65^Cu (*I* = 3/2) nuclei along the *g_z_* component are separately resolved for the central and terminal Cu^II^ centers in **2** (Figure 3; note the linewidth is too large to resolve the peaks from the different Cu isotopes). The relative intensities of the two components allow us to match the components to the two distinct copper sites.

The better resolved, frozen solution cw EPR spectra could be simulated^[^ ^30^ ^]^ without including exchange interactions between the Cu centers, and hence independent of the relative orientation of the ***g***‐matrices, using the spin Hamiltonian:

$$\begin{array}{ccc} \hat{H} & = & \mu_{B}\;{\hat{S}}^{Cu2}\cdot g^{Cu2}\cdot B+\sum \mu_{B}{\hat{S}}^{Cu1}\cdot g^{Cu1}\cdot B\\ & & +\;{\hat{S}}^{Cu2}\cdot A^{Cu2}\cdot {\hat{I}}^{Cu2}+\sum {\hat{S}}^{Cu1}\cdot A^{Cu1}\cdot {\hat{I}}^{Cu1} \end{array}$$

These simulations, with a 1:2 weighting of the Cu2 to Cu1 components, give *g*‐values of ***g*** (Cu2) = [2.06 2.08 2.30] and ***g*** (Cu1) = [2.07 2.10 2.36], and copper hyperfine interactions of *A*_z_ = 440 MHz (both sites; *A*_x,y_ unresolved) that are typical for the approximately tetragonal geometries of the Cu1 and Cu2 sites. The absence of any effect of Cu…Cu interactions in the cw EPR spectra shows that these interactions are significantly smaller than the experimental linewidths. Variable‐temperature studies of the solution spectra show the resolution of the two distinct Cu^II^ sites remains up to 50 K (Figure S5). At 100 K and above, the resolution of the two distinct Cu^II^ sites is lost. This is most likely due to some flexibility of the molecule.

Cw EPR measurements on **4**, as a powder and a 1 mM solution in dry Et_2_O, again give spectra typical of tetragonal Cu^II^ centers (Figures S6 and S7). Only one distinct Cu^II^ site is observed, consistent with the structure; little change was seen with temperature. Both the solution and powder spectra of **4** could be simulated using the ***g***‐matrix of the central Cu2 ion in **2** (Figures S6 and S7).

### 2.3 Pulsed EPR Spectroscopy

In order to probe the geometry of the supramolecule in the solution phase, **2** was studied by pulsed EPR. Echo‐detected field‐swept EPR spectra at X‐ and Q‐bands (0.2 mM in Et_2_O, 5.7 K) are consistent with cw EPR spectra (Figure S8). Inversion recovery and Hahn‐echo decay measurements at X‐band gave spin‐lattice relaxation and phase memory time constants of *T_1_* ≈ 1.5 ms and *T_m_* ≈ 800 ns, respectively (Figures S9 and S10); similar times were found at both the *g*_z_ and *g_x,y_* regions of the spectrum.

**Figure 4:**
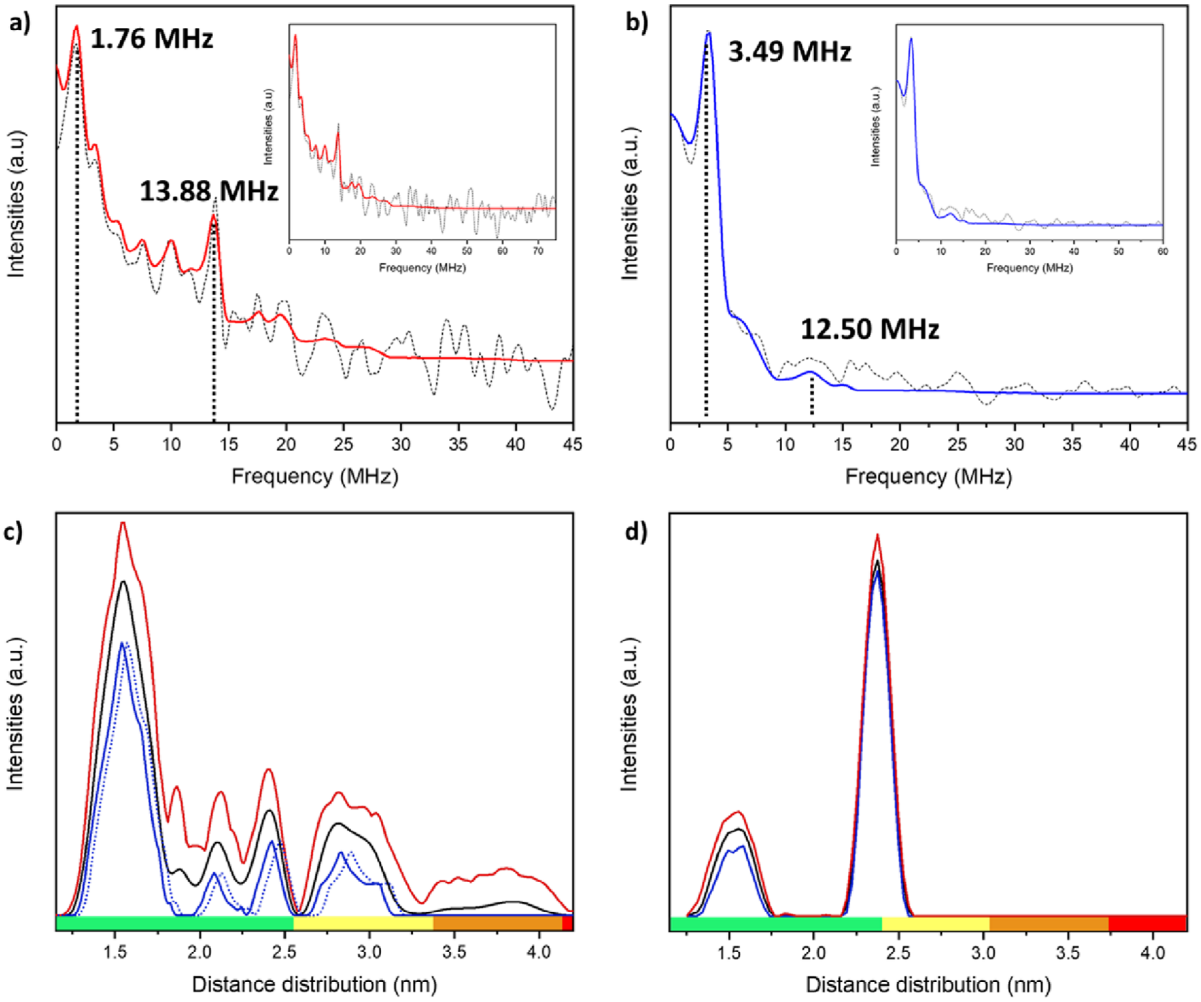
DEER measurements on **2** in 0.2 mM Et_2_O solution at 5.7 K, at (a, c) X‐ and (b, d) Q‐band, with the pump pulse at *g*_xy_. (a,b) Pake patterns, experimental (dotted black line) and fit using DeerAnalysis2022 (red/blue). (c, d) Distance distributions from Tikhonov regularizations and validation against background correction functions: mean probability (black), lower (blue) and upper (red) error estimates (mean plus or minus twice the standard deviation); color coding on the distance axis refers to reliability based on the maximum mixing time in the pulse sequence (see Supplementary Information). The dashed blue lines are the distance distributions corrected for the effective *g*‐values at the experimental *B*_0_ fields.

Four‐pulse Double Electron‐Electron Resonance^[^ ^31^ ^]^ spectroscopy (DEER; also known as PELDOR, Pulsed Electron Double Resonance) was performed at X‐ and Q‐band to probe the interactions between the Cu sites. DEER is a two‐frequency technique involving pump and detection pulses to address different spins in a pair: the refocused Hahn echo intensity of the detection spin is monitored as a function of the timing of the pump pulse. In initial X‐band experiments, the data analysis (see below and Supplementary Information) gave a distance distribution with a dominant peak at *ca*. 1.6 nm (Figure 4c) which agrees well with the short Cu1…Cu2 distances from the crystal structure and suggested a longer distance at around 3 nm which may correspond to the Cu1…Cu1 distance. However, the X‐band dipolar evolution time traces (Figure S11) were compromised by ^1^H ESEEM (electron spin echo envelope modulation) effects, which we could not remove satisfactorily by τ‐averaging. Given that the ^1^H Larmor frequency at X‐band (14.3 MHz at *B*_0_ = 336 mT) is close to the dipolar frequency expected for the short distance (a 15.7 Å distance would correspond to 13.4 MHz for *g* = 2.0, for *B*_0_ perpendicular to the Cu…Cu vector), we then measured DEER at Q‐band. At Q‐band the ^1^H Larmor frequency is shifted to a higher frequency (50.2 MHz at *B*_0_ = 1178 mT) and out of the frequency range of interest, thereby allowing the dipolar interaction frequency to be distinguished from ESEEM oscillations.

Initial Q‐Band DEER measurements were performed with the pump pulse at the Cu *g*_xy_ maximum (see Supporting Information for details). The dipolar evolution time trace exhibits clear oscillations to >1 µs (Figure S12). An initial analysis was performed via Tikhonov regularization of the background‐corrected and Fourier‐transformed dipolar evolution (Figure S12b), using DeerAnalysis2022 software.^[^ ^32^ ^]^ This gave a distance distribution (corrected for effective *g*‐values of the pump and detection pulse positions)^[^ ^33^ ^]^ with peaks at 1.6 and 2.4 nm (Figure 4d). The 1.6 nm distance agrees well with the short Cu1…Cu2 distance from the crystal structure (1.57 nm), and implies that the dominant distance from X‐band (Figure 4c) does have contributions from this interaction, although it may also have a contribution from ^1^H ESEEM.

The observation of the longer Cu…Cu distance in the Q‐band data (Figure 4d) corroborates the suggestion of a longer distance in the X‐band data and would be consistent with the gross supramolecular structure being retained in solution (rather than dissociating into dimetallic and monometallic fragments). However, the distance derived from the orientation‐independent DeerAnalysis kernel is significantly shorter than the Cu1…Cu1 distance from the crystal structure (3.1 nm). If we were to interpret this distance as resulting from a simple Cu1…Cu2…Cu1 bend away from the strictly linear geometry observed in the crystal, this would correspond to an angle of ca. 100° (treating the two Cu1…Cu2 arms as rigid), which is unfeasible. This is due to the neglect of orientation selective effects in the DEER experiment;^[^ ^34^, ^35^, ^36^ ^]^ analysis of the latter requires measurement at more than one *B*_0_ field. However, attempts to measure a trace on the *g*_z_ spectral region under the same conditions (Figure S12a) did not have sufficient signal‐to‐noise for a clear analysis.

Hence, we performed further Q‐band DEER measurements using an AWG source that allows the use of shorter and better‐defined rectangular pump pulses, giving greater excitation bandwidth and hence modulation depths (see Table S4 for experimental parameters; τ‐averaging was employed to suppress ^14^N ESEEM). These measurements gave sufficient signal‐to‐noise to probe both the *g*_xy_ and the *g*_z_ spectral regions necessary for orientation‐selective analysis. We observed artifacts in the time traces close to the zero‐time (Figure S13). These are due to uncancelled echo crossings arising from the coherent pump and detection pulses and imperfect phase cycling,^[^ ^37^ ^]^ hence the zero‐time region was replaced by a fitted second‐order polynomial. This has the effect of obscuring the region where higher frequency oscillations, corresponding to the shorter Cu1…Cu2 distance, would be observed. Hence, the time traces are dominated by oscillations due to the long Cu1…Cu1 distance, and the analysis below neglects the short Cu1…Cu2 distances. The latter are defined well by the initial Q‐band DEER measurements, which used separate, hence incoherent, pump and detection sources.

**Figure 5:**
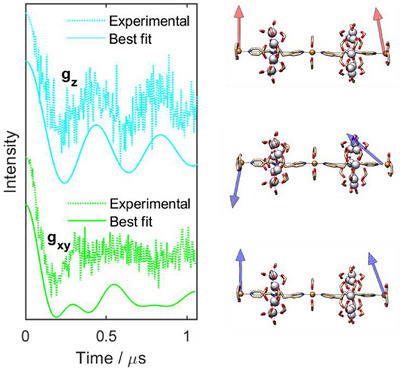
(left) Normalized experimental Q‐band DEER traces for **2** in 0.2 mM Et_2_O solution at 10 K (green and cyan traces correspond to detection at the copper *g_xy_* and *g_z_* spectral regions, respectively) with the corresponding simulated *g_z_* orientation fits. (right) Illustration of the three most abundant *g_z_* orientations (red arrows if Cu1‐Cu1 distance = 3.1 nm, blue arrows if Cu1‐Cu1 distance = 3.0 nm) appearing in the fit (accounting for 78% of the fit), overlaid with the simplified XRD structure, arranged from most abundant (top; 44%) to least abundant (bottom).

We first consider the orientations of the Cu1 ***g***‐matrices with respect to the Cu1…Cu1 vector. The oscillations in the time trace data are clearly at a higher frequency when measured at *g*_xy_ than at *g*_z_ (the first maxima in the time traces are observed at ca. 0.3 and 0.4 µs, respectively; Figures 5 and S13). In the high‐field limit, the dipolar interaction frequency (ω) is proportional to $\omega \propto g_{1}g_{2}\left(1-3\cos^{2}\theta \right)/r^{3}$ where *g*_1,2_ are the effective *g*‐values of the spins at the *B*_0_ of measurement, *r* is the inter‐spin separation, and θ is the angle between the *B*_0_ field and the inter‐spin vector. If the structure of **2** in the crystal was retained in solution, the local *g_z_* axes of the Cu1 sites would be parallel to the Cu1…Cu1 vector (Figure 2), and therefore detecting on *g_xy_* would be dominated by θ = 90° giving $\omega \propto g_{xy}^{2}/r^{3}$, while detecting at *g_z_* would be dominated by θ = 0° giving $\left|\omega \right|\propto 2g_{z}^{2}/r^{3}$. As such, we would detect a higher frequency at *g*_z_. Hence, the fact that the *g_xy_* trace has *higher* frequency oscillations than those for *g_z_* implies that the *g_z_* axes of the Cu1 sites have reoriented in the solution phase. To analyze these data, we calculated DEER traces for 874 orientations of the two Cu1 *g_z_* vectors (relative to each other and to the Cu1…Cu1 vector; Figure S14) and then fit this trace library to the experimental data using an iterative least‐squares fitting algorithm (see Supplementary Information for details).^[^ ^38^, ^39^ ^]^ We initially fixed the Cu1…Cu1 distance as 3.1 nm. With this model we could produce the *g*_xy_ and *g*_z_ time traces qualitatively; in order to better match the experimentally observed frequencies, it was necessary to allow for a minor population with a slightly shorter distance of 3.0 nm. We find that the best fits are dominated by conformations where the *g*_z_ axes of the Cu1 sites are near orthogonal to the Cu1…Cu1 vector (Figure 5). Hence, there is a gross reorientation of the ***g***‐matrices at Cu1 relative to the structure in the crystal. In the crystal, these sites have square‐pyramidal geometry (see above). It is possible that in the solution phase Cu1 remains square‐pyramidal but rotated such that one of the Cu‐O bonds becomes apical; this would match the geometry at the Cu sites in the crystal structure of **4**. A second possibility is that solvent Et_2_O molecules coordinate at Cu1, making the latter six‐coordinate, with the resulting Jahn‐Teller distortion axis *cis* to the pyridyl ligand. The necessity of including a minor population with a slightly shorter distance of 3.0 nm simply relates to conformational flexibility: any deviation from linearity of Cu1…Cu2…Cu1 will decrease the distance from that seen in the crystal structure. Note we observe flexibility of the molecule in the variable‐temperature cw EPR spectra (see above).

This model explains the importance of the orientation‐selective analysis. With the *g*_z_ axes perpendicular to the Cu…Cu vector but not necessarily coparallel to each other, then measuring at *g*_xy_ will be dominated by orientations of the Cu…Cu vector at around θ = 0° with respect to *B*_0_, because the *g*_z_ axes of the two Cu1 sites can be at any angle with respect to each other via rotation about the Cu‐N bonds. In contrast, θ = 90° would require the two *g*_z_ to be nearly coparallel, which is much less likely. In this case (θ = 0°), a frequency component of close to 3.5 MHz corresponds to twice the dipolar frequency, corresponding to a distance of ca. 3.1 nm. Neglecting orientation selection would assume a normal Pake pattern dominated by θ = 90° and give a shorter distance.^[^ ^33^ ^]^

## 3 Conclusion

We have shown that through the design of the thread, discrete rotaxane molecules can be used as a building block to produce a controlled Cu…{Ti_7_Ga}…Cu…{Ti_7_Ga}…Cu adduct containing two dissimilar spin *s* = ½ components. We have also shown that the extent of the oligomerization can be controlled by the steric demands of the thread, with a symmetric short thread giving a Cu…{Ti_7_Ga}…Cu adduct. This design would allow for the same structures to be achieved but using a different ring system that can be paramagnetic; any {Ti_7_M} ring would achieve the same outcomes. The {Cu(hfac)_2_} motif can also be changed to any other {M(hfac)_2_}, allowing a wide choice of spin systems in an ABABA array, which is appealing to both chemists and physicists to study; we are working toward these compounds. The results also show the power of using orientation‐selective DEER methods to provide information about the solution‐state structure not only in terms of conformational flexibility but also coordination changes at the terminal Cu centers. Using a combination of DEER experiments, both the short and long Cu…Cu separations could be detected and the relative orientations of the local ***g***‐matrices.

## Supporting Information

All data further supporting this study is provided in the Supplementary Information (SI) file accompanying this paper. The authors have cited additional references within the Supporting Information.^[^ ^40^, ^41^ ^]^

## Conflict of Interest

The authors declare no conflict of interest.

## Supporting information



Supporting Information

Supporting Information

## Data Availability

The data that support the findings of this study are available in the supplementary material of this article.
