# Supplementary material for: A [3]Rotaxane Containing {Ti7Ga} Rings Linking CuII: Synthesis, Structure, and Spectroscopic Studies
Source: Chemistry. 2025 Sep 30;32(28):e02530. doi: 10.1002/chem.202502530 (PMC13411496; doi:10.1002/chem.202502530)
Supplement: Supplementary file 1 — Supporting Information [file CHEM-32-e02530-s002.pdf]

Supporting Information

A [3]Rotaxane Containing {Ti_7Ga} Rings Linking Cu^{II}: Synthesis, Structure and Spectroscopic Studies

Selena J. Lockyer,[a] Lubomir Loci,[a] George F. S. Whitehead,[a] Inigo J. Vitorica-Yrezabal,[a] Grigore A. Timco,[a] Alice M. Bowen,[a] Eric J. L. McInnes,[a] and Richard E. P. Winpenny.[a]

[a] School of Chemistry and Photon Science Institute, The University of Manchester, Oxford Road, Manchester M13 9PL, U.K.

CONTENTS:

1. Experimental Section. S2

{Ga_3} Triangle Synthesis. S2

Organic Thread Synthesis. S2

Heterometallic Rotaxane Synthesis. S3

Extended Hybrid Heterometallic Rotaxane Synthesis. S5

2. Crystallography. S7

Table S1: Crystallographic data for **2** and **4**. S8

3. Structures. S9

4. Continuous-Wave Electron Paramagnetic Resonance Measurements. S10

5. Advanced Pulse EPR Techniques. S13

Electron Spin Relaxation Measurements. S14

Double Electron-Electron Resonance (DEER) Measurements. S15

DEER Orientation Analysis. S20

1. Experimental Section

General remarks: All starting reagents and materials used were sourced from Sigma-Aldrich and/or Fluorochem. Unless stated otherwise, all reagents and solvents were used without further purification. The compounds were synthesized and crystallized under nitrogen atmosphere in anhydrous solvents, using standard Schlenk line techniques. Chemical shifts are reported in parts per million (ppm) from low to high frequency and referenced to the residual solvent resonance. ESI mass spectrometry and microanalysis were carried out by the services at The University of Manchester.

Synthetic methods:

1 {Ga_3} triangle synthesis:

1.1 [$Ga_3O(O_2C^tBu)_7(CH_3CN)$] 0.5 $^tBuCO_2H$ was prepared as previously described.[25]

2 Organic Thread Synthesis:

Reductive amination - Schiff base condensation method:

2.1 Thread **A**, (**$PyCH_2NHCH_2CH_2Py$**):

A solution of 4-pyridinecarboxaldehyde (0.65 g, 6.1 mmol) and 4-(2-aminoethyl)pyridine (0.70 g, 6.1 mmol) in methanol (30 mL) was refluxed for 4 hr under an N_2 atmosphere, then stirred at room temperature for 3 hr. Excess $NaBH_4$ (0.95 g, 25.0 mmol) was added and the reaction mixture was stirred for 5 hr. The reaction was quenched with water (20 mL) and the residue was extracted with dichloromethane (3 x 25 mL). The dichloromethane extract was dried ($MgSO_4$) and the solvents evaporated under reduced pressure. An orange oil solution was produced; yield: 0.73 g, 57%. ^1H NMR (400 MHz, 293K, $CDCl_3$) δ 8.15-8.46 (m, 4H), 7.20 (d, 2H), 7.12 (d, 2H), 3.80 (s, 2H), 3.44 (m, 1H), 2,92-2.87 (t, 2H), 2.82-2.75 (t, 2H); ^{13}C NMR (400 MHz, 293K, $CDCl_3$) δ 149.74 (Ar), 149.20 (Ar), 148.97 (Ar), 147.30 (Ar), 124.15 (Ar),

122.88 (Ar), 52.45 (CH_2), 49.32 (CH_2), 35.74 (CH_2); ESI MS *m/z* (relative intensity) 214.2 $[M+H]^+$, 236.1 $[M+Na]^+$.

2.2 Thread **B**, (**$PyCH_2NHCH_2Py$**):

To a solution of 4-pyridinecarboxaldehyde (3.24 g, 30 mmol) and 4-(2-aminomethyl)pyridine (2.92 g, 27 mmol) in methanol (30 mL), NaBH4 (1.08 g, 28.5 mmol) was added slowly. The solution was stirred at RT for 20 hours before being quenched with HCl (20 drops). The solvents were evaporated under reduced pressure to produce a concentrated oil. Next, H_2O (60 mL) was added and the solution was made basic with the addition of solid K_2CO_3, and stirred for 30 mins. The product was extracted with $CHCl_3$ (3 x 20 mL) and washed with Sat. Aq. $NaHCO_3$ (50 mL). The $CHCl_3$ extract was dried ($MgSO_4$) and the solvents evaporated under reduced pressure. The light orange oily solution was purified by column chromatography, isolating the product as the first fraction using 3% MeOH in CHCl3; yield: 0.90 g, 17%. ^1H NMR (400 MHz, 293K, $CDCl_3$) δ 8.57 (d, 4H), 7.31 (d, 4H), 3.85 (s, 4H); ^{13}C NMR (400 MHz, 293K, $CDCl_3$) δ 150.00 (Ar), 148.87 (Ar), 122.90 (Ar), 51.79 (CH_2); ESI MS *m/z* (relative intensity) 200.1 $[M+H]^+$.

3 Heterometallic Rotaxane Synthesis: {(R,R'NH$_2$)[Ti$_7$GaO$_8$(O$_2$C^{t}Bu)$_{16}$]}:

3.1 Synthesis of **1 {($PyCH_2NH_2CH_2CH_2Py$)[$Ti_7GaO_8(O_2C^tBu)_{16}$]}**:

Me_3CCO_2H (36 g, 356 mmol), thread **A** (0.5 g, 2.3 mmol) and pivalic anhydride (1 mL, 4.93 mmol) were heated to 50 °C under an N_2 atmosphere until a clear solution was present. $[Ga_3O(O_2C^tBu)_7(CH_3CN)]$ 0.5 $^tBuCO_2H$ (1 g, 0.98 mmol) and toluene (20 mL) were added and refluxed at 140 °C for 30 minutes and a clear solution was obtained. The solution was cooled to 70 °C and titanium(IV) isopropoxide (5.0 mL, 16.9 mmol) was added. The reaction

mixture was then stirred while refluxing for 24 hours at 140 °C. The toluene was removed by distillation, and the residue cooled to 70 °C and MeCN (100 mL) was added. The solution was left to cool to room temperature while stirring. The precipitate was collected by filtration, washed with MeCN (4 x 25 mL) and acetone (2 x 5 mL), and then extracted into hexane (50 mL). Solvents were removed under reduced pressure to produce a dry light orange powder, isolated as four fractions. Yield: 2.20 g, 40%. ESI MS *m/z* (relative intensity) 2365 $[M+H]^+$, 2387 $[M+Na]^+$. Elemental analysis %: calc. for $C_{93}H_{160}GaN_3O_{34}Ti_7$: Ga 2.95, Ti 14.17, C 47.23, H 6.82, N 1.78; found: Ga 2.67, Ti 15.08, C 45.84, H 6.83, N 1.47.

3.2 Synthesis of **3 $\{(PyCH_2NH_2CH_2Py)[Ti_7GaO_8(O_2C^tBu)_{16}]\}$**:

Me_3CCO_2H (36 g, 356 mmol), Thread **B** (0.5 g, 2.5 mmol) and pivalic anhydride (1 mL, 4.93 mmol) were heated to 50 °C under an N_2 atmosphere until a clear solution was present. $[Ga_3O(O_2C^tBu)_7(CH_3CN)]$ 0.5 $^tBuCO_2H$ (1 g, 0.98 mmol) and toluene (20 mL) were added and refluxed at 140 °C for 30 minutes and a clear solution was obtained. The solution was cooled to 70 °C and titanium(IV) isopropoxide (5.0 mL, 16.9 mmol) was added. The reaction mixture was then stirred while refluxing for 24 hours at 140 °C. The toluene was removed by distillation, and the residue cooled to 70 °C and MeCN (100 mL) was added. The solution was left to cool to room temperature while stirring. The precipitate was collected by filtration, washed with MeCN (4 x 25 mL) and acetone (2 x 5 mL), and then extracted into hexane (50 mL). Solvents were removed under reduced pressure to produce a dry light-yellow powder. Yield: 3.50 g, 60%. ESI MS *m/z* (relative intensity) 2352 $[M+H]^+$, 2350 $[M-H]^-$. Elemental analysis %: calc. for $C_{92}H_{158}GaN_3O_{34}Ti_7$: Ga 2.97, Ti 14.25, C 47.00, H 6.77, N 1.79; found: Ga 2.76, Ti 15.08, C 47.34, H 7.05, N 1.38.

4 Extended hybrid heterometallic rotaxane synthesis:

4.1 Synthesis of **2** **$\{(PyCH_2NH_2CH_2CH_2Py)_2[Ti_7GaO_8(O_2C^tBu)_{16}]_2[Cu(hfac)_2]_3\}$**:

Under an N_2 atmosphere, a solution containing **1** (0.33 g, 0.14 mmol) and copper(II) hexafluoroacetylacetonate hydrate (0.1 g, 0.21 mmol) dissolved in diethyl ether (20 mL) and toluene (5 mL), was stirred for 20 minutes. Light green crystals suitable for single crystal X-ray diffraction were grown via slow evaporation over a slow flow of N_2 over 24 hours. The crystals were separated by filtration and washed with toluene. Yield: 1.9 g (44%). Elemental analysis %: calc. for $C_{216}H_{326}Cu_3F_{36}Ga_2N_6O_{80}Ti_{14}$: Cu 3.07 Ga 2.25, Ti 10.79, C 42.54, H 5.29, N 1.35; found: Cu 2.98, Ga 1.76, Ti 11.67, C 43.12, H 5.24, N 1.36.

4.2 Synthesis of **4** **$\{(PyCH_2NH_2CH_2Py)[Ti_7GaO_8(O_2C^tBu)_{16}][Cu(hfac)_2]_2\}$**:

Under an N_2 atmosphere a solution containing **3** (0.160 g, 0.068 mmol) and copper(II) hexafluoroacetylacetonate hydrate (0.065 g, 0.136 mmol) dissolved in dry diethyl ether (10 mL) and dry toluene (5 mL) was stirred for 20 minutes. Light green crystals suitable for single crystal X-ray diffraction were grown via slow evaporation over a slow flow of N_2 over 24 hours. The crystals were separated by filtration and washed with toluene. Yield: 0.11 g (50%). Elemental analysis %: calc. for $C_{112}H_{162}Cu_2F_{24}GaN_3O_{48}Ti_7$: Cu 3.84 Ga 2.11, Ti 10.13, C 40.69, H 4.95, N 1.27; found: Cu 3.77, Ga 2.29, Ti 10.38, C 43.84, H 4.82, N 1.10.

(a)

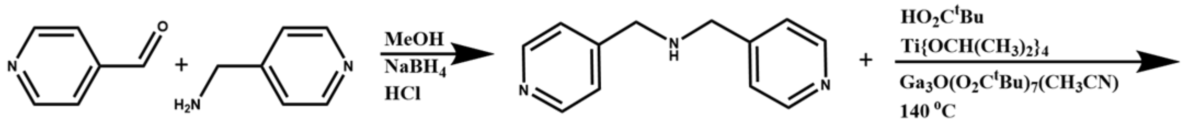

(b)

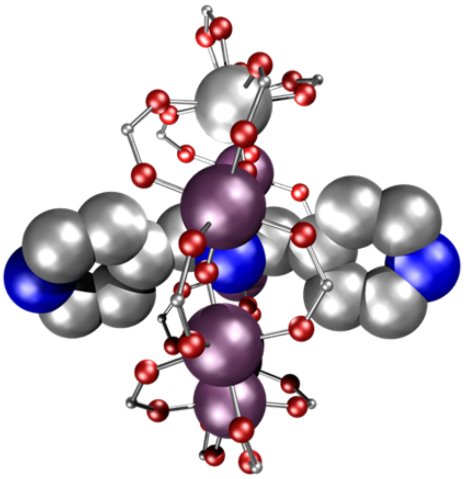

Figure S1. (a) The synthesis and (b) the structure of **3**. Atoms within the thread **B**H shown with van der Waals radii. All other atoms shown as ball-and-stick representations. Atom colors: blue (N), red (O), grey (C), mauve (Ti), silver (Ga). ^{t}Bu groups and hydrogens omitted for clarity.

2. Crystallography

Data Collection:

X-ray diffraction data for compound **2** was collected at a temperature of 150 K using a Rigaku FR-X with Cu-Kα radiation equipped with a Hypix 6000HE detector, equipped with an Oxford Cryosystems nitrogen flow gas system. Data was measured using CrysAlisPro suite of programs. X-ray diffraction data for compound **4** was collected at Diamond Light Source beamline (I19) using Zr-L edge wavelength (λ = 0.6889 Å) radiation at a temperature of 100 K. Data were collected using GDA.

Crystal structure determinations and refinements:

X-Ray data were processed and reduced using the CrysAlisPro suite of programs. Absorption correction was performed using empirical methods (SCALE3 ABSPACK) based upon symmetry-equivalent reflections combined with measurements at different azimuthal angles.[40] The crystal structure was solved and refined against all F^2 values using the SHELXL and Olex 2 suite of programmes.[41]

CCDC 2477655 and 2477658 contain the supplementary crystallographic data for **2** and **4**, respectively, for this paper. These data can be obtained free of charge *via* www.ccdc.cam.ac.uk/conts/retrieving.html (or from the Cambridge Crystallographic Data Centre, 12 Union Road, Cambridge CB21EZ, UK; fax: (+44)1223-336-033; or deposit@ccdc.cam.ac.uk).

Table S1. Crystallographic Data for Compounds 2 and 4		
	**2**	**4**
Identification code	rrepw209	m-sjl640A-3-c1_
Empirical formula	$C_{224}H_{342}Cu_3F_{36}Ga_2N_6O_{95}Ti_{14}$	$C_{125.5}H_{186}Cu_2F_{24}GaN_3O_{50.5}Ti_7$
Formula weight	6323.31 g mol^{-1}	3532.86 g mol^{-1}
Temperature/K	150.00(10) K	100.00(10) K
Crystal system	tetragonal	monoclinic
Space group	$P4_22_12$	$P2_1/c$
a/Å	27.1953(4)	18.7462(2)
b/Å	27.1953(4)	30.9988(4)
c/Å	21.3433(4)	58.0682(6)
α/°	90	90
β/°	90	90.4200(10)
γ/°	90	90
Volume/Å^3	15785.2(6)	33743.1(7)
Z	2	8
ρ_{calc}g/cm^3	1.330	1.391
μ/mm^{-1}	4.075	0.754
F(000)	6538	14584
Crystal size/mm^3	0.019	0.421 × 0.251 × 0.074
Radiation	CuKα (λ = 1.54184)	synchrotron (λ = 0.6889)
2Θ range for data collection/°	4.596 to 155.05	2.888 to 41.598
Reflections collected	58269	205290
Independent reflections	16251 [R_{int} = 0.0485, R_{sigma} = 0.0529]	38117 [R_{int} = 0.960, R_{sigma} = 0.0628]
Data/restraints/parameters	16251/886/1138	38117/21178/4459
Goodness-of-fit on F^2	1.015	1.345
Final R indexes [I>=2σ (I)]	R_1 = 0.0667, wR_2 = 0.1851	R_1 = 0.1069, wR_2 = 0.3208
Final R indexes [all data]	R_1 = 0.0858, wR_2 = 0.2051	R_1 = 0.1287, wR_2 = 0.3491
Largest diff. peak/hole / e Å^{-3}	1.09/-0.54	0.99/-0.58
Flack parameter	-0.007(3)	N/A

3. Structures

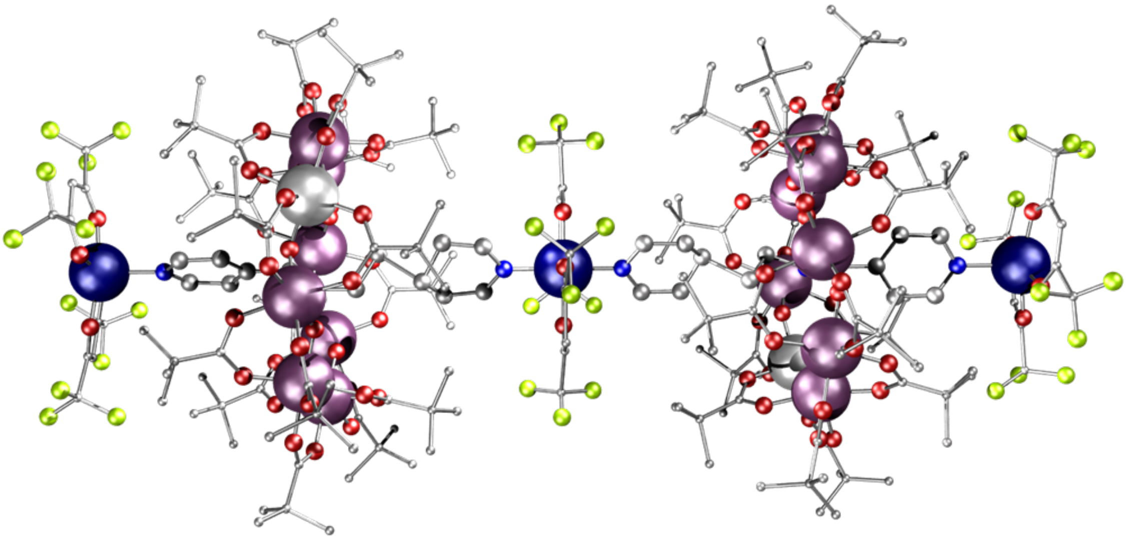

Figure S2. Crystal structure **2**. Atom colors: blue (N), red (O), grey (C), mauve (Ti), silver (Ga) and yellow (F). Hydrogens omitted for clarity.

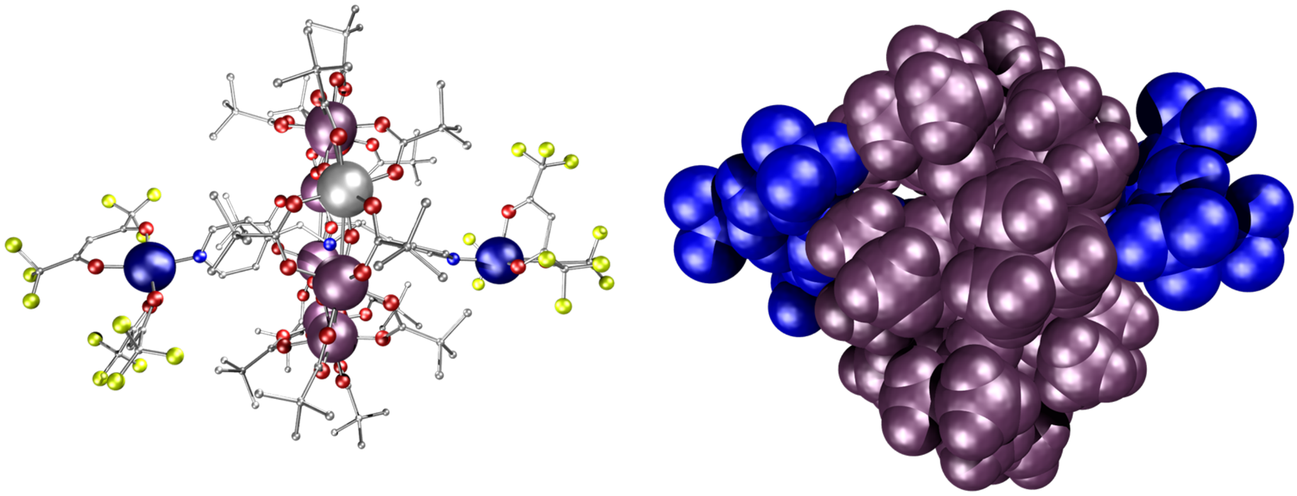

Figure S3. Left: Crystal structure **4**. Atom colors: blue (N), red (O), grey (C), mauve (Ti), silver (Ga), yellow (F). Hydrogens omitted for clarity. Right: Space-filling view with with threads and [Cu(hfac)$_2$] units shown in blue, and {Ti7Ga} rings in mauve.

4. Continuous-Wave Electron Paramagnetic Resonance Measurements

Continuous-wave (cw) X-band (~9.5 GHz) and Q-Band (~34 GHz) EPR spectra were recorded with a Bruker EMX580 spectrometer; the data was collected on polycrystalline powders and as a solution in dry Et_2O, at 5 K (unless otherwise stated) using liquid helium cooling. All continuous-wave spectra were field-corrected using a Bruker 'Strong Pitch' standard (g = 2.0028) and all powder samples were checked for any polycrystalline nature, by measuring multiple random rotations.

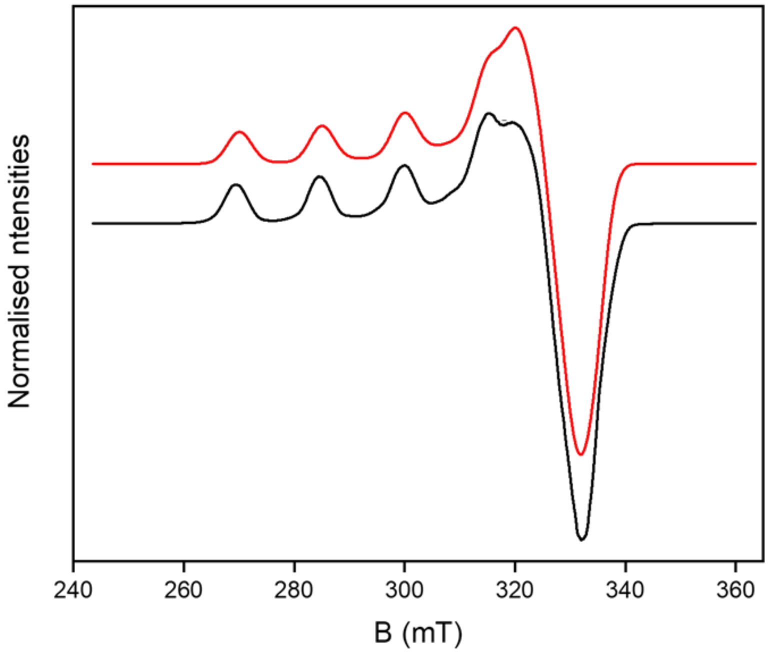

Figure S4. CW EPR X-Band (*ca.* 9.5 GHz) spectra of **2** as a powder. Experimental measurement at 5 K (black) and simulation (red). Simulation[29] parameters as per manuscript.

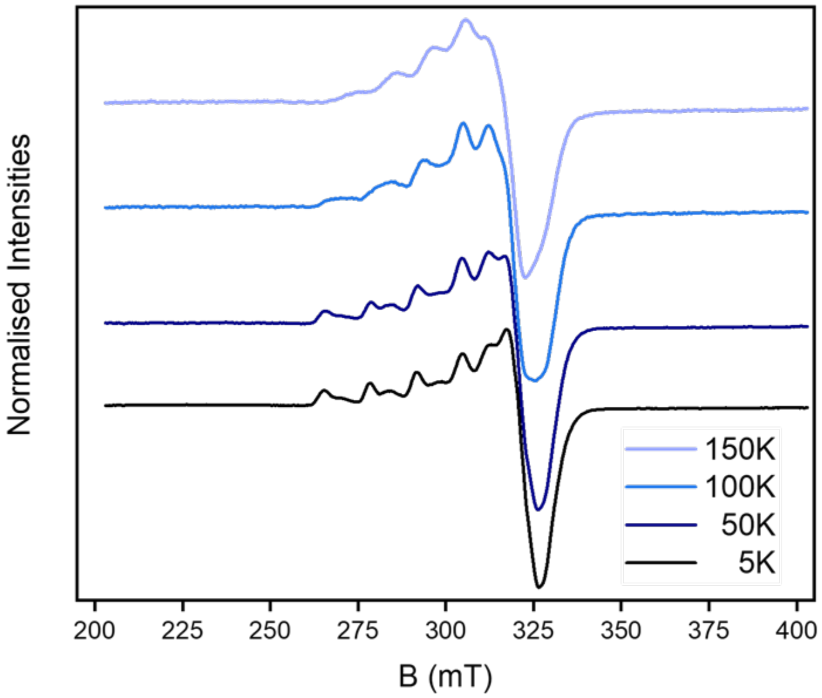

Figure S5. CW EPR X-Band (*ca.* 9.5 GHz) spectra of **2** as a 1 mM solution in dry and degassed Et_2O. Experimental temperatures provided in figure.

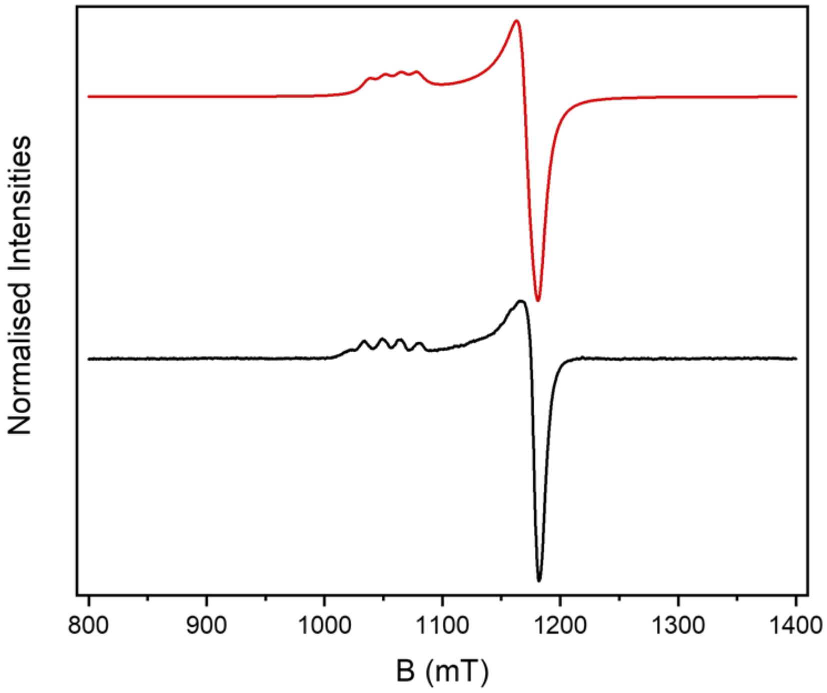

Figure S6. CW EPR Q-Band (*ca.* 34 GHz) spectra of **4** as a 1 mM solution in dry and degassed Et_2O, at 5K. Experimental (black line) and simulation (red line). Simulation parameters provided in manuscript.

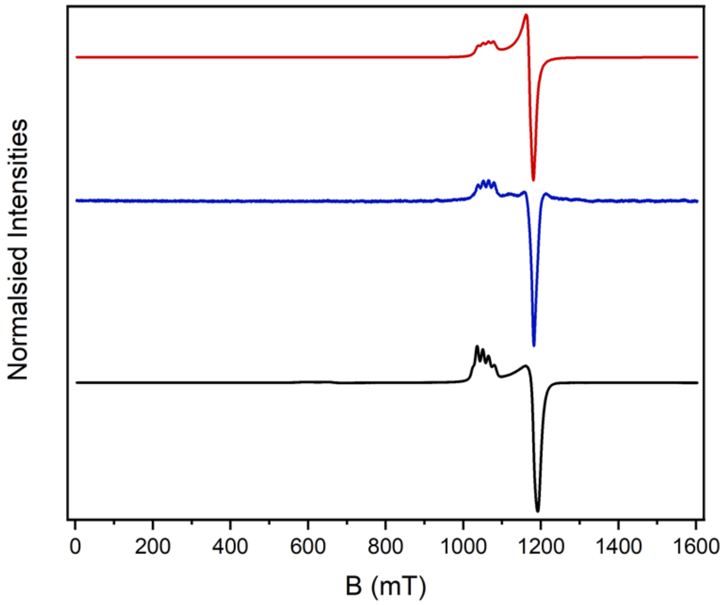

Figure S7. CW EPR Q-Band (*ca.* 34 GHz) spectra of **4** as a powder. Experimental at 5K (black line), 290 K (blue line) and simulation (red line). Simulation parameters provided in the manuscript.

5. Pulsed EPR Techniques

5.1 Electron Spin Relaxation Measurements:

Pulsed X-band (~9.7 GHz) EPR data were collected on a Bruker ELEXSYS E580X FT spectrometer. The data were collected from a solution of dry and degassed Et_2O (0.2 mM) at 5.7 K (unless otherwise stated). Pulsed Q-band (34 GHz) EPR data were collected on a Bruker ELEXSYS E580Q FT spectrometer. The data were collected from a solution of dry and degassed Et_2O (0.2 mM) at 3 K (unless otherwise stated).

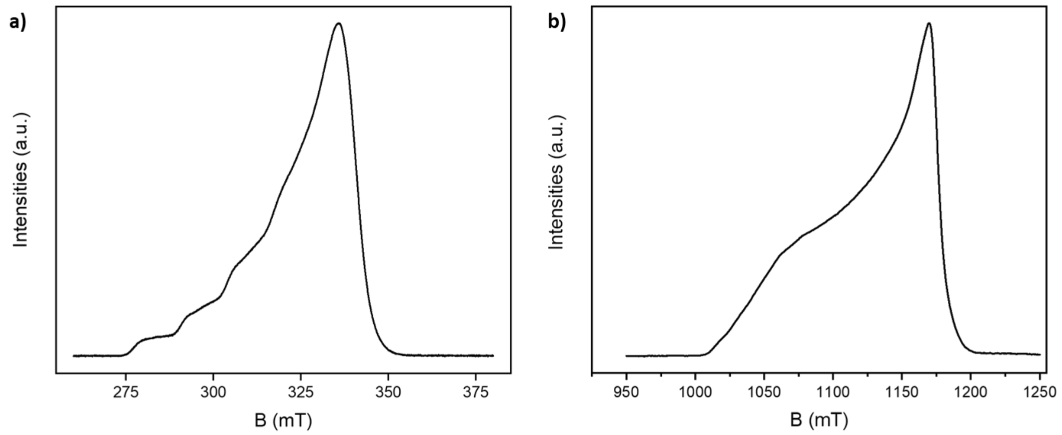

Figure S8. Echo Detected Field Sweep (EDFS) for **2** as a 0.2 mM concentration using a standard Hahn echo sequence π/2-τ-π-τ-echo at: a) X-Band (*ca.* 9.7 GHz), 5.7 K with 16 and 32 ns pulses for π/2 and π, respectively and τ = 200 ns. b) Q-Band (*ca.* 34 GHz) 3 K with 16 and 32 ns pulses for π/2 and π, respectively and τ = 300 ns.

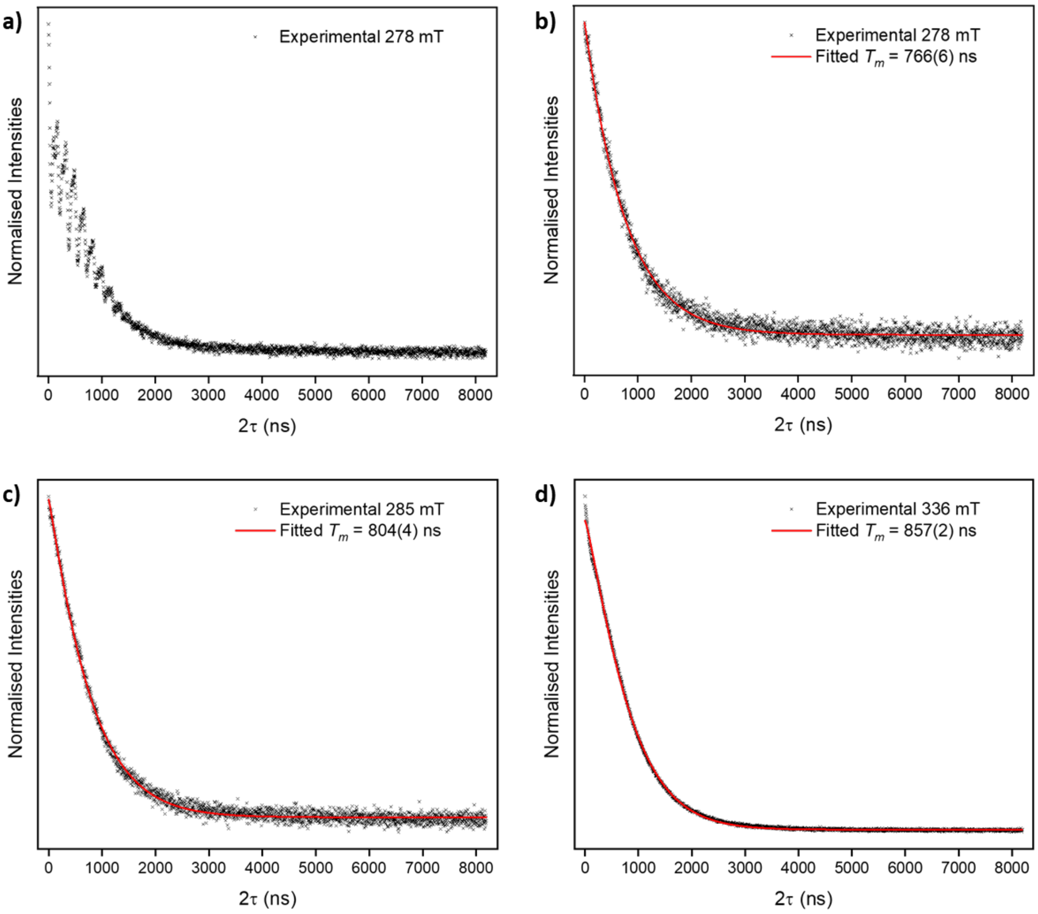

Figure S9. Phase memory times (T_m) for **2** observed at 278 mT (a,b), 285 mT (c) and 336 mT (d). A π/2-τ-π-τ-echo sequence was used with 16 (π/2) and 32 (π) ns pulses with a tau of 100 ns for the top left (a) with ESEEM, and with longer pulses of 100 (π/2) and 200 (π) ns pulses with a tau of 200 ns, to suppress the ESEEM (b-d). The echo decays were fit to an exponential decay with the form $I(2\tau) = I(2\tau_0)\exp(-2\tau/T_m)$.

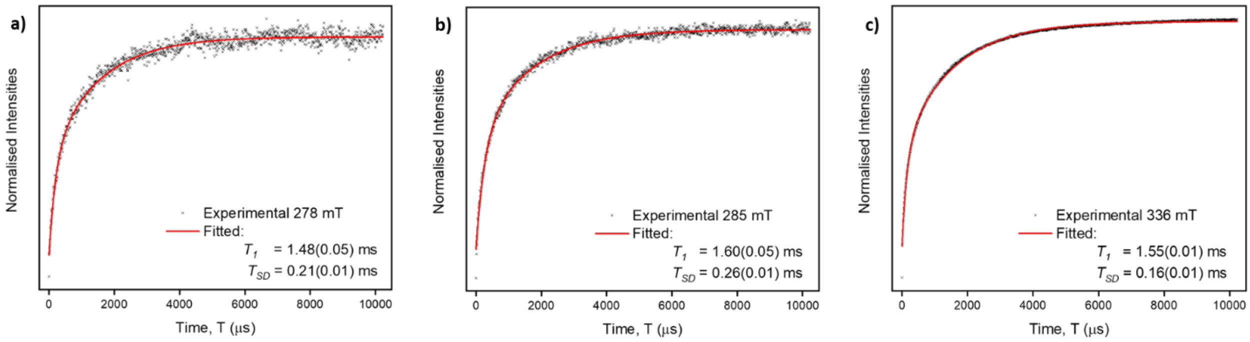

Figure S10. Spin lattice relaxation times (T_1) for **2**, observed at 278 mT a), 285 mT b) and 336 mT c). Measurements were performed with a sample concentration of 0.2 mM at 5.7 K. A π-T-π/2-τ-π-τ-echo sequence was used with 16 (π/2), 32 (π) ns pulses and a τ of 1000 ns. The echo decays were fit to an exponential decay with the form $I(\tau) = I_1(\tau_0)\exp(-\tau/T_1)+I_2(\tau_0)\exp(-\tau/T_{SD})$

5.2 Double Electron-Electron Resonance (DEER):

For X-Band a four-pulse DEER sequence was performed for **2**, with the ELDOR pulse at the Cu g_{xy} maximum (B_0 = 338 mT, 9.697 GHz) and the observer pulse positioned at +100 MHz. Pulse lengths were 12 and 24 ns for $^{\pi}/_2$ and π, respectively, with an ELDOR pulse of 20 ns and τ_1 = 200 ns and τ_2 = 1300 ns.

For Q-Band a four-pulse DEER sequence was performed for **2**, with the ELDOR pulse (33.975 GHz) at the Cu g_{xy} and g_z maxima (B_0 = 1201.7 mT and 1091.4 mT, respectively), and the observer pulse positioned at -75 MHz in each case. Pulse lengths were 16 and 32 ns for $^{\pi}/_2$ and π, respectively, with an ELDOR pulse of 40 ns and τ_1 = 150 ns and τ_2 = 1800 ns.

Attempts to measure DEER experiments on **4** showed no oscillations in the time traces. This is possibly due to the shorter Cu…Cu distance (14.3 Å) compared to the short Cu1…Cu2 distance in 2 (15.7 Å).

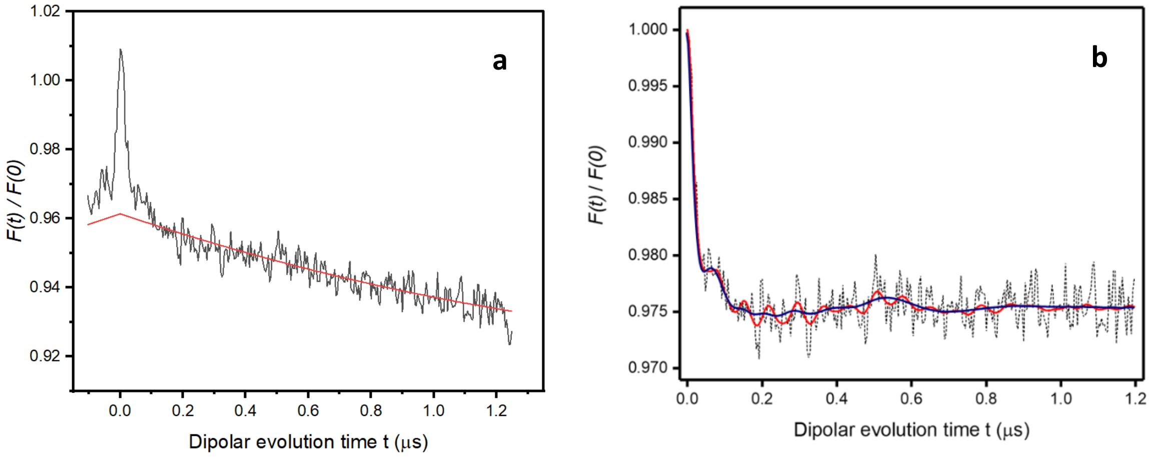

Figure S11. Dipolar evolution traces of a 4-pulse DEER sequence for **2**, as a 0.2 mM Et_2O solution at X-Band (*ca.* 9.7 GHz), at 5.7 K. (a) Raw trace (black line) with the background (red line) fitted using DeerAnalysis.[32] (b) Background-corrected trace: experimental (black dotted line), fits using DeerAnalysis (solid red line and navy blue line, for total fitting and omitting proton ESEEM, respectively).

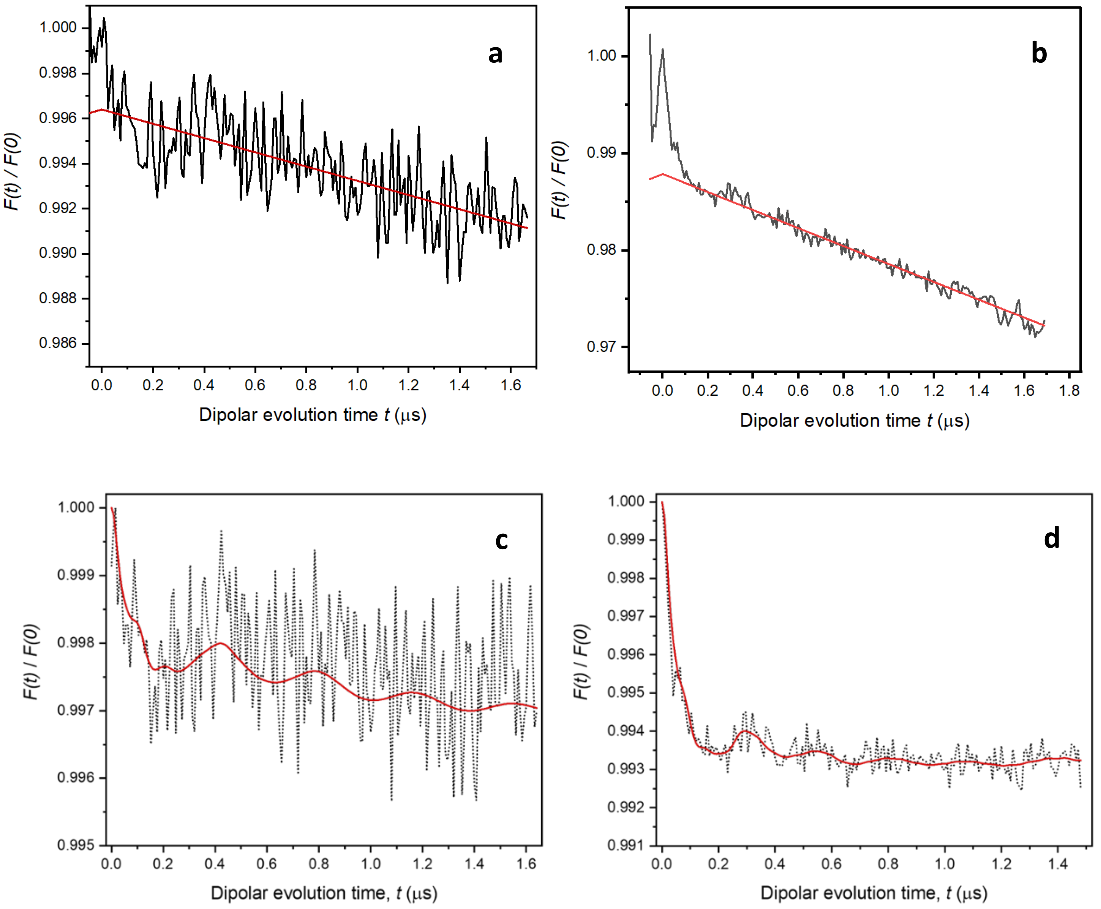

Figure S12. Dipolar evolution traces of a 4-pulse DEER sequence for **2**, as a 0.2 mM Et_2O solution at Q-Band (*ca.* 34 GHz) and 5 K. (a, b) Raw DEER traces (solid black line) with their backgrounds (solid red line) fitted using DeerAnalysis.[32] (c, d) Background-corrected DEER traces; experimental (black dotted line), fitting using DeerAnalysis (solid red line). At field positions (a, c) B_0 = 1091.4 mT and (b, d) B_0 = 1201.7 mT.

**Table S2: DEER Parameters (X-band)**		
Compound:		**2**
Sample conditions:	Concentration	0.2 mM
	Tube diameter	4 mm
	Sample volume	0.1-0.2 ml
	Cryoprotectant	Liquid N_2
	Freezing procedure	Flash-freezing
	Deuteration	*None*
	Solvent	Diethyl ether
	Temperature	5.7 K
Instrumentation:	Spectrometer	Bruker Elexsys E580
	Resonator	MD5
EPR Parameters:	Pump π-pulse length	20 ns
	Det π/2-pulse length	12 ns
	Det π-pulse length	24 ns
	Field	3380 G
	Pump frequency	9.697 GHz
	Det frequency	9.797 GHz
	Pump-det offset	-100 MHz
	Pump pulse shape	Rectangular
	Det pulse shape	Rectangular
	τ_1 length	200 ns
	τ_2 length	1300 ns
	Shot repetition time	3060 μs
	Time increment	4 ns
	Shots per point	10
	Number of averages	15250
Nuclear modulation averaging procedure:	Tau-averaging (τ_1, ^{1}H)	Time step: 8 ns<br>Number of τ_1 to average: 8
Measures to reduce multi-spin effects:		*None as multi-spin effects assumed to be negligible at X-band (low inversion efficiencies)*
Data parameters:	Modulation depth	2.4 %
	SNR (wrt mod depth)	8.0 (no units)
	Zero-time offset	200 ns

**Table S3: DEER Parameters (Q-band)**		
Compound:		**2**
Sample conditions:	Concentration	0.2 mM
	Tube diameter	3 mm
	Sample volume	0.1-0.2 ml
	Cryoprotectant	Liquid N_2
	Freezing procedure	Flash-freezing
	Deuteration	*None*
	Solvent	Diethyl ether
	Temperature	5 K
Instrumentation:	Spectrometer	Bruker Elexsys E580
	Resonator	QT2
EPR Parameters:	Pump π-pulse length	40 ns
	Det π/2-pulse length	16 ns
	Det π-pulse length	32 ns
	Pump frequency	33.975 GHz
	Det frequency	33.900 GHz
	Pump-det offset	+75 GHz
	Pump pulse shape	Rectangular
	Det pulse shape	Rectangular
	τ_1 length	150 ns
	τ_2 length	1800 ns
	Shot repetition time	20400 μs
	Time increment	8 ns
	Shots per point	*See below*
	Number of averages	*See below*
	Pulse generation	MPFU and ELDOR
Nuclear modulation averaging procedure:	Tau-averaging (τ_1, ^{14}N)	Time step: 32 ns<br>Number of τ_1 to average: 8
Measures to reduce multi-spin effects:		*None as multi-spin effects assumed to be negligible at Q-band (low inversion efficiencies)*
Data parameters:	Modulation depth	*See below*
	SNR (wrt mod depth)	*See below*
	Zero-time offset	100 ns

Detection region	**Cu g_{xy}**	**Cu g_z**
Field / G *	12017	10914
Number of averages	4288	13593
Shots per point	2	2
Modulation depth / %	0.7	0.3
SNR wrt MD / no units	12	2.1

** The reported field values are obtained directly from the instrument reading and do not take into account the significant field offset of the Q-band instrument at the time of measurement; the true magnetic field values are approximately 350 G lower than the reported values.*

5.3 DEER Orientation Analysis:

**Table S4: DEER Orientation Parameters**		
Compound:		**2**
Sample conditions:	Concentration	0.2 mM
	Tube diameter	1.6 mm
	Sample volume	0.1-0.2 ml
	Cryoprotectant	Liquid N_2
	Freezing procedure	Flash-freezing
	Deuteration	*None*
	Solvent	Diethyl ether
	Temperature	10 K
Instrumentation:	Spectrometer	Bruker Elexsys E580
	Resonator	QT2
EPR Parameters:	Pump π-pulse length	28 ns
	Det π/2-pulse length	14 ns
	Det π-pulse length	28 ns
	Pump frequency	33.94 GHz
	Det frequency	34.015 GHz
	Pump-det offset	-75 MHz
	Pump pulse shape	Rectangular
	Det pulse shape	Rectangular
	τ_1 length	120 ns
	τ_2 length	*See below*
	Shot repetition time	5100 μs
	Time increment	4 ns
	Accumulation time	*See below*
	Number of averages	*See below*
	Pulse generation	AWG
Nuclear modulation averaging procedure:	Tau-averaging (τ_1, ^{14}N)	Time step: 36 ns<br>Number of τ_1 to average: 8
Measures to reduce multi-spin effects:		*None as multi-spin effects assumed to be negligible at Q-band (low inversion efficiencies)*
Data parameters:	Modulation depth	*See below*
	SNR (wrt mod depth)	*See below*
	Zero-time offset	40 ns

Detection region	**Cu g_{xy}**	**Cu g_z**
Field / G *	11990	10940
τ_2 / ns	1400	1200
Accumulation time / hours	16	22.5
Number of averages	12	20
Shots per point	20	20
Modulation depth / %	0.7	0.6
SNR wrt MD / no units	20	10

** The reported field values are obtained directly from the instrument reading and do not take into account the significant field offset of the Q-band instrument at the time of measurement; the true magnetic field values are approximately 350 G lower than the reported values.*

The raw 4-pulse DEER traces collected at Q-band for orientation analysis contained distortions near the zero-time, likely due to uncancelled echo crossings arising from coherent pump and detection microwave pulses. To remedy this, the zero-time region was replaced by a fitted second-order polynomial to remove the artefacts; both the original and the modified sets of DEER traces are illustrated in Figure S13. Fitting regions: (-16)-(-8) ns and 52-72 ns for g_{xy}, (-32)-(-24) ns and 64-84 ns for g_z.

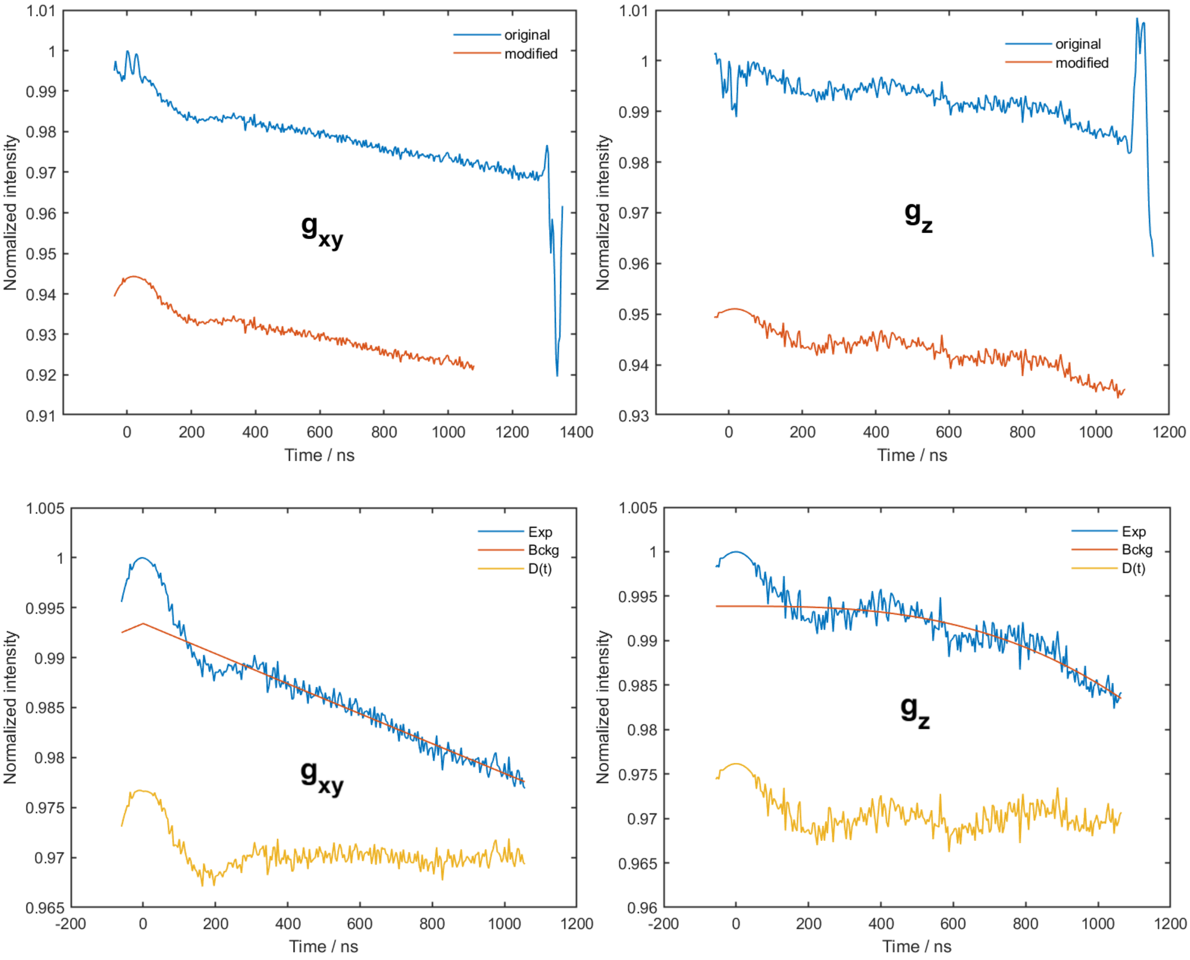

Figure S13. Normalized Q-band DEER dipolar evolution traces on **2** (solution as above), measured at the copper g_{xy} (left, field = 1199 mT) and g_z (right, field = 1094 mT) spectral regions, shown alongside their modified analogues (top left = g_{xy}, top right = g_z) where the zero-time region was fitted with a polynomial function. The modified traces (bottom, blue lines) then had any remaining background contributions (bottom, red lines) removed via DeerAnalysis to give the final background-corrected traces (bottom, yellow lines).

DEER traces were calculated for variable orientations of the terminal Cu1 g_z orientations using the geometric model illustrated in Figure S14. The molecule as a whole was assumed to be rigid, with two possible inter-copper distances (3.00 and 3.10 nm) for each pair of g_z orientations. Due to the symmetry of the molecule and the invariance of EPR spectra with respect to inversion, only g_z vectors pointing to the surface of an octant of a sphere were considered (see Figure S14).

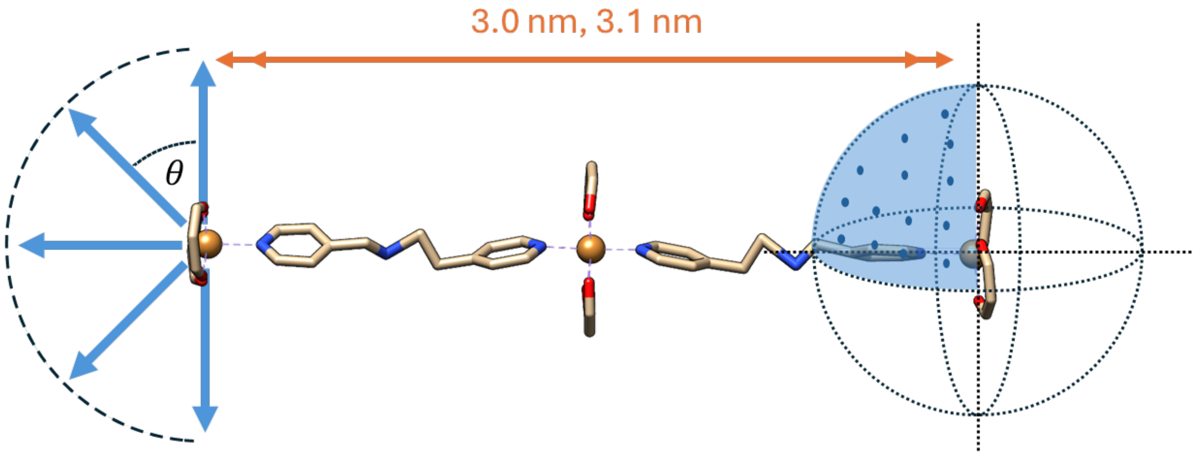

Figure S14. Illustration of the geometric model used to simulate DEER for a variety of g_z orientations. For one terminal copper (left), g_z vectors (blue arrows) were varied by rotation about the copper in the plane of the page (θ = 0° to 180° in 10° increments). For the other terminal copper (right), 46 equidistant g_z orientations were simulated over an octant-sphere surface (shaded blue with dots) for each orientation of the left terminal copper g_z. Finally, each g_z orientation pair was simulated for two copper-copper distances (3.0 nm and 3.1 nm).

The least-squares fitting algorithm used to fit the traces was reported previously for a set of [4]rotaxane systems.[39] The algorithm was allowed to run for 50 fitting cycles; by this point, the least-squares difference between the simulation and the experimental data was no longer decreasing with additional cycles. The abundances of the 3 most abundant conformations found from the fit (from most to least abundant) are as follows: 1st – 44%; 2nd – 22%; 3rd – 12%.
